# Temporal and inter-individual changes in the integrated biochemical condition of the gonads of female swordfish (*Xiphias gladius*) from the Southeastern Pacific Ocean

**DOI:** 10.7717/peerj.15524

**Published:** 2023-06-06

**Authors:** Fabián Guzmán-Rivas, Juan Ortega, Sergio Mora, Patricio Barría, Rodrigo Riera, Ángel Urzúa

**Affiliations:** 1 Facultad de Ciencias, Programa de Doctorado en Ciencias con mención en Biodiversidad y Biorecursos, Universidad Católica de la Santísima Concepción, Concepción, Biobío, Chile; 2 Instituto de Fomento Pesquero, Talcahuano, Biobío, Chile; 3 Instituto de Fomento Pesquero, Valparaíso, Valparaíso, Chile; 4 Departamento de Biología, Universidad de Las Palmas de Gran Canaria, Las Palmas de Gran Canaria, España; 5 Departamento de Ecología, Facultad de Ciencias, Universidad Católica de la Santísima Concepción, Concepción, Biobío, Chile; 6 Centro de Investigación en Biodiversidad y Ambientes Sustentables (CIBAS), Universidad Católica de la Santísima Concepción, Concepción, Biobío, Chile

**Keywords:** Sexual maturity, Body size, Oocyte, Gonads, Energy reserves, Highly migratory fish species, Seasonality

## Abstract

The integrated biochemical condition (IBC) of gonads is closely related to the reproductive success of highly migratory marine species. The IBC of gonads can be influenced not only by size and/or age, but also by environmental conditions. Here, female swordfish, *Xiphias gladius*, that migrate to temperate regions with a marked seasonality (*e.g*., the Southeastern Pacific Ocean, SEPO) were compared in relation to the IBCs (lipids, proteins, glucose and, fatty acid profiles) of their gonads; individuals with two body size ranges and distinct degrees of sexual maturity were evaluated, and considered as: small and/or virginal (SV: <170 cm lower jaw fork-length (LJFL), oocyte size (OS) <0.08 mm) *vs* large and/or maturing females (LM: >190 cm LJFL, OS >0.133 mm). This comparison was conducted in two environmentally contrasting seasons (winter *vs* spring). Our results showed that the gonadosomatic index (GSI) was significantly higher in LM than SV. Lipid contents varied significantly between seasons and body sizes. The highest lipid concentrations were recorded in the spring in large females. No significant differences were found when comparing the protein and glucose contents of the two evaluated seasons or body size ranges of the studied females. In turn, the fatty acid (FA) profiles of female gonads significantly varied for both seasons and body size ranges. A high content of saturated fatty acids (SFAs), monounsaturated fatty acids (MUFAs) and poly-unsaturated fatty acids (PUFAs) were recorded in female gonads in the spring. The SFAs C16:0 and C18:0, the MUFA C18:1*n*9, and the essential PUFA C22:6*n*3 were the main contributors to the observed differences between spring and winter. These results could be used as indicators of the nutritional condition and health status of swordfish individuals. Hence, the IBC of female swordfish gonads have great potential to aid in estimating survival rates and stock abundances of this species. The integration of this information constitutes an asset in fishery management models with an ecosystem approach.

## Introduction

The integrated biochemical condition (IBC) of the gonads is one of the most important physiological biomarkers recently used in fishery assessment with an ecological approach (Hochachka & Somero, 2002; Marshall Adams, Burtis & Beauchamp, 1985). In the case of marine fish species with migratory cycles, the IBC of the gonad plays an important role, not only for reproductive success, but also for the survival of the first stages of ontogeny (Hochachka & Somero, 2002; Kjørsvik, Mangor-Jensen & Holmefjord, 1990). This is because the IBC of the gonad is composed mainly of essential biochemical constituents and/or bioenergetic reserves (lipids, proteins and carbohydrates: Betsy & Kumar (2020), Eliassen & Vahl (1982)), which during the reproductive process are transferred from the gonad to the oocytes during the process of oogenesis (Bucholtz et al., 2013; Lubzens et al., 2010). The subsequent utilization of these biochemical elements also has high implications for latent and/or carry-over effects (Harrison et al., 2011), which can occur throughout the different phases of early fish ontogeny: from embryogenesis, the development of the vitelline larva and until the beginning of the first exogenous feeding (Boros, Sály & Vanni, 2015; Costalago et al., 2012). The IBC includes a high number of processes, such as the biosynthesis, absorption, accumulation, and transport of pivotal biomolecules that are crucial for the posterior differentiation and growth of eggs. Hence, any factor interacting even subtly with any of the above-mentioned processes may harm the quality and biochemical condition not only of the eggs, but also of the vitelline larvae (Dabrowski, 1986; Harrison et al., 2011).

In general, the biochemical condition of the gonad is related to the amount and type of food available in the environment (*i.e*., food supply), which in marine environments of temperate latitudes (*e.g*., the Southeastern Pacific Ocean, SEPO), can vary at the individual level (depending on the size and/or age) and also on a temporal scale (throughout the seasons of the year). In this context, recent studies indicate that the general bioenergetic condition of adult individuals of marine invertebrates and fishes in temperate environments vary depending on food availability and water temperature (Guzmán-Rivas et al., 2021; Lazo-Andrade et al., 2021b; Rosa et al., 2010). For example, in the case of highly migratory fishery resource species (swordfish, sharks, squids) from the SEPO (Claramunt et al., 2009; Francis, Holdsworth & Block, 2015; Yu et al., 2015), it has been described that adult individuals of jumbo squid (*Dosidicus gigas*) and swordfish (*Xiphias gladius*) have a high and/or optimal biochemical condition during spring-summer, when warm water temperatures and high plankton productivity (Lazo-Andrade et al., 2021a; Quispe-Machaca, 2019) predominate in the environment. Consistently, this superior biochemical condition coincides with the beginning of its reproductive period.

In the case of some highly migratory species (*e.g*., *D. gigas*), it has been described that the amount of bioenergetic reserves varies, not only seasonally and/or temporally, but also intra-individually (*i.e*., among organs and/or tissues: liver, gonad, muscle) and among individuals with different degrees of sexual maturity (Quispe-Machaca et al., 2021, 2022). In the case of the swordfish *X. gladius*, it has recently been found that the biochemical constituents of the muscle of adult individuals (*i.e*., sexually mature) varies seasonally (Lazo-Andrade et al., 2021a), though it is unknown whether these temporal variations occur in other organs that are important for reproduction (*e.g*., the gonad) and among females with different degrees of sexual maturity (virginal *vs* maturing females).

The bioenergetic condition of the gonad can be explained by some intrinsic natural factors related to the biology of mature females, including their size and/or age. Former studies have shown that middle-aged females produced better quality eggs than females spawning for the first time (Leschen, Grady & Valiela, 2006) because middle-aged females can incorporate higher amounts of proteins and fatty acids in their oocytes (Kjørsvik, Mangor-Jensen & Holmefjord, 1990). Moreover, environmental factors, such as temperature, salinity, and oxygen concentration can also directly influence the state of the embryos, affecting their metabolic rates and/or their energy demand and thus leading to variations in size, weight, and biochemical condition (Evans & Claiborne, 2005; Johnston & Leggett, 2002; Migaud et al., 2013).

Currently, different criteria have been used to evaluate gonad quality, such as (a) morphometric criteria, including size and weight and (b) biochemical criteria, including lipid, protein, and glucose contents, as well as fatty acid profiles (Hasek & Felder, 2005; Vázquez et al., 1994). Unfortunately, most observations regarding the biochemical criteria of gonads have been conducted in cultured species (Bobe, 2015), while scarce information is available on wild pelagic species. This information gap is accentuated in highly migratory marine species (*e.g*., tunas, sharks, swordfish, and billfish) (Abascal et al., 2009), which are more complex to study considering their size and/or age (Poisson & Fauvel, 2009), and their trans-oceanic migrations (Abascal et al., 2009).

The swordfish *Xiphias gladius* (Linnaeus, 1758), considered as a highly migratory fish, inhabits surface waters over 13 °C in the Pacific and Atlantic Oceans from 50°N to 50°S (Mejuto & García-Cortés, 2014; Nakamura, 1985; Stoehr et al., 2018). It is considered a key species and top predator in the marine food web (Stillwell & Kohler, 1985; Zambrano-Zambrano et al., 2019), feeding on other pelagic (*e.g*., horse mackerel, sardine, and sawfish; Clarke et al. (1995), Letelier et al. (2009), Abid et al. (2018)) and demersal species (*e.g*., squid, hake, squat lobsters, and myctophid fishes; Zambrano-Zambrano et al., 2019), in addition to supporting important fisheries (Ibáñez, González & Cubillos, 2004). In the SEPO, this species is in a state of full exploitation (SUBPESCA, 2022). Particularly, in the Chilean coast *X. gladius* is under a scientific monitoring program and is commercially captured from April to November (“cold period”) by the industrial and artisanal fishing industry, as a target species or as by-catch with an average of 2,256 tons (Barría et al., 2020) landed for 2018. In the warm period (December–March) swordfish migrate to equatorial zones of warm waters (~25 °C) of SEPO, where reproduction and spawning occur (DeMartini, Uchiyama & Williams, 2000; Donoso et al., 2003). In turn, its minimum allowable catch size (measured as lower jaw fork-length, LJFL) registered for swordfish on the Chilean coast is close to 100 cm of LJFL (Barría et al., 2020; SUBPESCA, 2022). The minimum capture size was established based on the criteria of size and age of the individual, which in this case corresponds to approximately 1 year of life (ca. 12 months) (Barría et al., 2020; SUBPESCA, 2022).

The life-history traits and reproductive characteristics of *X. gladius* consist of: (i) several reproductive events characterized by partial spawning and homogeneous maturation of the gonad (Claramunt et al., 2009), (ii) high fecundity, (iii) a large body size of over 180 cm at maturity (Barría et al., 2016; Froese, 2004), (iv) maturation at 4 years of age (DeMartini, Uchiyama & Williams, 2000), and (v) a maximum observed longevity of 16 years (Cerna, 2009; DeMartini et al., 2007). Altogether, these life-history traits can provide swordfish with a competitive advantage and adaptive plasticity to temperate coastal marine habitats. For example, the size and/or age of females may promote a cascading/carry-over effect on the bioenergetic condition of gonads, which can later determine the reproductive success and stability of the population. This information can serve as an important aid in stock evaluation, especially for recruitment and population growth modeling (Hixon, Johnson & Sogard, 2013; Kjesbu et al., 2009).

Provided that *X. gladius* is a species with late sexual maturation, a high longevity, and a large body size that spawns and reproduces multiple times, we hypothesized that not only the age, size, and/or degree of sexual maturity of females influence the bioenergetic condition of their gonads, but also the seasonal environmental changes in the SEPO within its reproductive cycle. Therefore, to test this hypothesis, we herein evaluated the biochemical condition (measured as the content of lipids, proteins, glucose, and fatty acids (FAs)) of the gonads of two body size ranges and/or distinct degrees of sexual maturity (small and/or virginal *vs* large and/or maturing females) female swordfish from two contrasting seasons (winter *vs* spring) in the SEPO. Additionally, energetic implications were discussed within an ecosystem approach for the evaluation and management of this fishery resource.

## Materials and Methods

### Ethical declaration

This research was conducted in accordance with the Act on Welfare and Management of Aquatic Animals, and they comply with the current Chilean animal care and manipulation legislation of the fishery resources (SUBPESCA, 2022). Consequently, to avoid the pain of the animals during their capture and processing, they were put to sleep with a cold shock (Law 20.380, Ministry of Health, Chile) (Robb & Kestin, 2002).

### Sampling procedures

**Figure 1:**
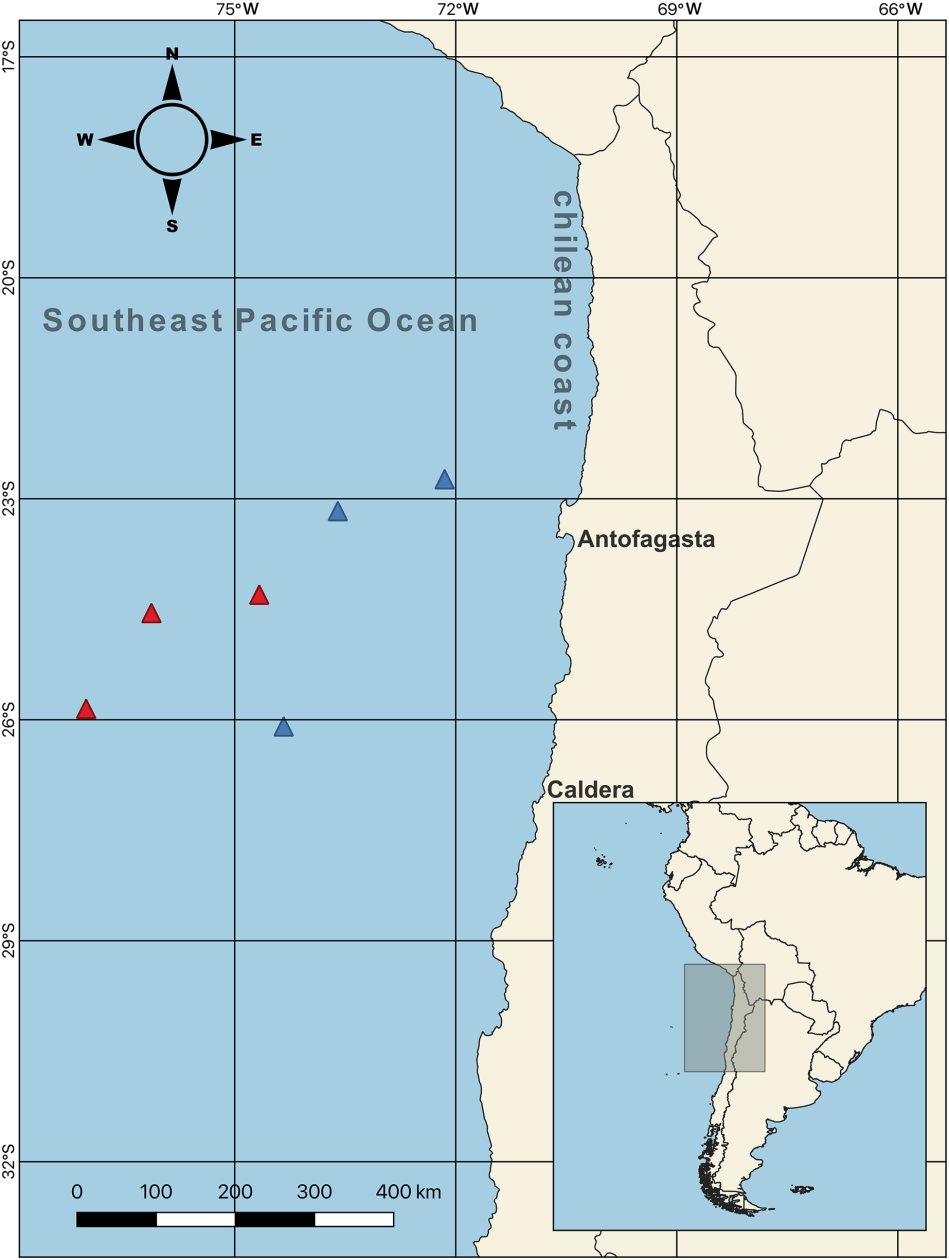
Sampling points of females swordfish. The color-coded triangles indicate exact points of extraction for each swordfish (*i.e*., red = winter sampling (June–August 2017); blue = spring sampling (October–December 2017)), the samples were taken off the Chilean coasts (between Antofagasta and Caldera, indicated with their respective names on the map) in the Southeast Pacific Ocean. Total *N* = 44; all samples were obtained from the six sampling points indicated on the map.

Female swordfish (*N* = 44; size range, based on their lower jaw fork-length: 128–262 cm) were collected from the SEPO off the Chilean coast between Antofagasta and Caldera (23° 10′ S, 73° 36′ W; 26° 05′ S, 74° 20′ W; 22° 43′ S, 72° 09′ W; 24° 18′ S, 74° 40′ W; 25° 51′ S, 77° 01′ W; 24° 33′ S, 76° 07′ W) (Fig. 1, map) during the seasonal operational period of the fishing fleet (capture allowed; SUBPESCA, 2022), which coincided with the cold periods corresponding to two seasons of the year 2017, austral winter (July–August) and austral spring (October–November). As previously mentioned, during this cold period swordfish inhabits predominantly cold waters off the Chilean coast, subsequently in the months of the warm period (*i.e*., between December–March) swordfish migrate towards equatorial zones of warm waters of SEPO. Particularly, the winter and spring seasons present environmentally contrasting conditions (*i.e*., sea surface temperature and chlorophyll-a) in the SEPO. We used QGIS v3.22 software to create the map of the sampling points. Three long-liner vessels from the Chilean artisanal fleets: “Puerto Lindo”, “Vama II”, and “Arauco II”, were used for this purpose with the help of scientific observers from the Instituto de Fomento Pesquero (IFOP) (The Fishing Development Institute). After measuring the LJFL (in cm), individual females were separated into two body size ranges (indicated by the lower jaw fork-length: LJFL), and based on first size at sexual maturity: 180 cm LJFL (Barría et al., 2016; Froese, 2004); as follows, small and/or virginal-SV: <170 cm LJFL, *N* = 22; and large and/or maturing-LM: >190 cm LJFL, *N* = 22. Collected females were then weighed on a precision balance (Precisa, 120A) (*i.e*., total weight, TW, in kg). Their complete gonads were extracted, weighed on the same precision balance, cleaned, and stored in airtight boxes with dry ice to maintain a cold temperature. Subsequently, these samples were transported to the IFOP-Talcahuano to determine the parameters indicative of sexual maturity in female swordfish.

### Environmental parameters

Data on sea surface temperature (SST) and chlorophyll *a* (mg m^3^) (as a proxy of productivity and/or food availability) were obtained from NASA’s GIOVANNI database (NASA, 2017). These environmental data were downloaded for the sampling year (2017) and sampling area off the Chilean Pacific coast, between Antofagasta (23° 10′ S, 76° 36′) and Caldera (25° 51′ S, 77° 01′ W).

### Criteria indicative of female sexual maturity (LFJL, OS, GSI)

Three criteria were used to determine the degree of sexual maturity of females: (i) size of individuals, below or above the size at first sexual maturity described for this species (Barría et al., 2016; Froese, 2004) (<170 cm LJFL: virginal; >190 cm LJFL: maturing females), (ii) oocyte size (OS) (DeMartini, Uchiyama & Williams, 2000) (<0.08 mm: virginal; >0.133 mm: maturing females), (iii) gonadosomatic index (GSI) (Bolger & Connolly, 1989; Eyo, Ekanem & Jimmy, 2014) (<0.44: virginal; >0.45 maturing females).

The entire gonads were extracted from the females, measured and weighed on a digital scale (Precisa model 120A) following the methodology described by Poisson & Fauvel (2009). Considering that *X. gladius* presents a gradually homogeneous maturation of the gonad (Marisaldi et al., 2020; Poisson & Fauvel, 2009), tissue cuts of high precision were taken from the central area of the gonad to extract the oocytes, measure their size, and determine the stage of development for details of methods see Poisson & Fauvel (2009). Additionally, a piece (~80 mg of wet weight) from the central part of the gonad was extracted, stored in labeled vials, and preserved at −80 °C for posterior analyses of the integrated biochemical conditions at the Hydrobiological Resources Laboratory of the Universidad Católica de la Santísima Concepcion, Chile.

The OS measurements (diameter, length in mm) were carried out using a stereomicroscope (Euromex-Holland NexiusZoom) with a calibrated eyepiece, a micrometer, and an attached camera (CMEX 5 Pro). Images were digitized using the ImageFocus 4.0 software (Fig. S1). In turn, the stage of gonad development was determined following the macro-classification criteria for teleost fish proposed by Brown-Peterson et al. (2011), and then using the detailed and appropriate classification for swordfish proposed by DeMartini, Uchiyama & Williams (2000). Finally, the GSI was calculated as gonad weight/total body weight × 100 (Bolger & Connolly, 1989; Eyo, Ekanem & Jimmy, 2014).

### Integrated biochemical condition of the gonad

The samples were dried for 48 h in a lyophilizer (FDU-7012 Operon). The dry weights (DW, ±0.01 mg) were then determined using a precision scale (Precisa model 120A). To estimate the bioenergetic condition of the gonads, the proximate biochemical composition comprising lipids, proteins, glucose, and energy content, as well as the fatty acid profiles, were calculated from 50 mg DW of the gonad samples. All of the examined gonads were at the same stage of maturity; and all gonad samples were taken from well-preserved areas of the gonad tissue.

### Total lipid contents

The lipid contents of gonads were estimated following the method described by Folch, Lees & Sloane Stanley (1957). The samples were placed in 15 mL vials containing 5 mL of dichloromethane-methanol (2:1) and incubated in an ultrasonic bath with ultrapure water (Milli-Q) at 6 °C for 15 min. Each sample was mixed with 4 mL of potassium chloride (at 0.88%), homogenized in a vortex (Select Vortexer, mod. SBS100-2) for 15 s, and then centrifuged (Boeco, mod. S-8; Boeco, Akron, OH, USA) at 6 °C and 1,500 rpm for 5 min. The precipitate of each sample was extracted into a previously weighed vial and dried with ultrapure nitrogen gas in a sample concentrator to evaporate the solvent (Glass Col, mod. 109A YH-1; Glas-Col, LLC, Terre Haute, IN, USA). Finally, the dried samples were weighed (DW, ±0.01 mg) on a precision scale (Precisa, mod. 120A) and the concentration of lipids was obtained by subtracting the weight of the empty vial from the weight of the vial containing the sample. The lipid extract was preserved at −80 °C in dichloromethane-methanol (2:1) containing 0.01% butylated hydroxytoluene for further analyses of fatty acid compositions and profiles.

### Protein content

The total protein content of gonads was determined using the method proposed by Lowry et al. (1951), with modifications for microplate reading. Three reagents (S, A, and B) were utilized in the Bio-Rad DC Protein Assay Kit. A dried 50 mg sample of gonad tissue was diluted in 500 μL of ultrapure water (Milli-Q) and then homogenized. Five μL of the homogenized gonad sample, together with 200 μL of the B reagent and 25 μL of the A’ reagent (*i.e*., a mix of 20 μL of the S reagent and 1 mL of the A reagent), were transferred to a 96-well microplate. The samples were vortexed for 15 s in a Select Vortexer (mod. SBS100-2) and then incubated in the microplates for 15 min at room temperature prior to the measurements of absorbance. The absorbance was measured in a spectrophotometer at a wavelength of 750 nm (BioTek, mod. ELx808; BioTek, Winooski, VT, USA). The concentration of each sample was then obtained using a calibration curve for total proteins. This curve was attained by diluting different concentrations of bovine serum albumin (500-0111; Bio-Rad, Hercules, CA, USA).

### Glucose content

The glucose content of gonads was estimated following the method described by Tietz (1995). The gonad samples were rehydrated by adding 500 µL of distilled water in 1.5 mL Eppendorf vials. The content was homogenized (Scilogex, mod. D160; Scilogex LLC, Rocky Hill, CT, USA) at 3,000 rpm for 2 min. Samples were analyzed using a glucose kit (Spinreact), with a standard for glucose and a working reagent (WR) that reacts with the organic compound glucose. A micropipette (AxyPet®) was used and 10 μL of the standard was pipetted into a 1.5 mL Eppendorf tube containing 1 mL of WR. Eppendorf tubes were incubated at room temperature (18 °C) for 30 min. A microplate reader spectrophotometer (BioTek, mod. Elx808; BioTek, Winooski, VT, USA) was used to read 250 μL of each sample (Eppendorf tube) at a wavelength of 490 nm. The glucose content was estimated using the (Tietz, 1995) equation, in which the percentage of glucose is based on the dry weight [% glucose = (mg of glucose * 100)/sample dry weight (in mg)].

### Energy content

The energy content of the gonad tissue was measured in Joules (J) and estimated using the energy bioequivalence of the quantified biochemical components with the following conversion coefficients: 1 mg lipids = 39.54 J, 1 mg proteins = 23.69 J, and 1 mg glucose = 17.15 J (Urzúa et al., 2012; Winberg, 1971). Total energy was considered as the sum of the energy contributed by each of these biochemical components.

### Fatty acid profiles

Fatty acid (FA) profiles were determined using the methods presented by Cequier-Sánchez et al. (2008) and modified by Urzúa & Anger (2011). In short, fatty acid methyl esters (FAMEs) were measured using the total lipid extracts previously used for the estimations of total lipid contents. Total lipid extracts were esterified using methanolic sulfuric acid incubations at 70 °C for 60 min in a Thermo-Shaker (MRC model DBS-001). Fatty acids were then rinsed using 6 mL of n-hexane and concentrated using a sample concentrator to evaporate the solvent (Glass Col, model 109A YH-1). The measurement of FAMEs was performed using a gas chromatograph (Agilent, model 7890A; Agilent Technologies, Santa Clara, CA, USA), at a set temperature, equipped with a DB-225 column (J&W Scientific: 30 m in length, 0.25 internal diameter, and a 0.25 mm film). Using chromatograph’s software (Agilent ChemStation), individual FAMEs were identified by means of known standard FAs of marine origin (certificate material, Supelco 37 FAME mix 47885-U; Urzúa et al. (2013), Malzahn et al. (2007)) and quantified by means of the response factor to the internal standard (*i.e.*, 23:0 FA added prior to transmethylation).

### Statistical analysis

Statistical analyses were performed using standard methods (Lê, Josse & Husson, 2008; Sokal & Rohlf, 1995; Zuur, Leno & Smith, 2007) in SigmaStat 4.0 (Systat Sofware Inc.San Jose, USA), PRIMER 6.0, and R (*i.e*., FactorMineR; R Core Team, 2015), with significance at a 95% confidence level (*P* < 0.05). Additionally, SIGMAPLOT 12.0 (Systat Software Inc., San Jose, CA, USA) was used to generate figures. The normality of data distribution was evaluated with the Kolmogorov-Smirnov test, and homogeneity of variance was tested using Levene’s statistics. Tukey *post-hoc* tests were performed when significant differences were found between female sizes and seasons. A two-way ANOVA with fixed factors (*i.e*., season (winter *vs* spring) and female size, with two body size ranges and/or distinct degrees of sexual maturity (small and/or virginal-SV *vs* large and/or maturing-LM)) was used to determine variations in the biochemical composition and energy content. Non-parametric tests, namely PERMANOVA, PCoA, and SIMPER were used to evaluate the differences in the FA composition and FA profiles of gonads in SV *vs* LM females collected in two distinct seasons (winter *vs* spring).

## Results

### Environmental parameters

Sea surface temperatures (SSTs) and chlorophyll *a* showed marked seasonal differences. Lower SSTs were measured in the winter (17.37 ± 0.38 °C) and gradually increased throughout the spring-summer (20.75 ± 0.81 °C) (Table 1). In turn, the chlorophyll *a* concentrations showed a different profile, with a lower chlorophyll *a* content in the autumn (0.18 ± 0.04 mg m^3^) and a peak in the winter (0.28 ± 0.06 mg m^3^) (Table 1).

**Table 1:** Monthly variations in environmental factors (*i.e*., sea surface temperature in °C and chlorophyll *a* concentration in mg m^3^) from 2017 off the northern Chilean coast.

Parameters	January	February	March	April	May	June	July*	August*	September*	October*	November*	December
Temperature(°C)	21.37 ± 0.64	22.31 ± 0.51	22.89 ± 0.94	22.15 ± 0.77	20.94 ± 0.43	18.96 ± 0.46	18.37 ± 0.38	17.72 ± 0.72	17.23 ± 0.66	17.38 ± 0.51	17.96 ± 0.43	18.58 ± 1.01
Chlorophyll(mg m^3^)	0.15 ± 0.04	0.15 ± 0.03	0.14 ± 0.04	0.18 ± 0.03	0.22 ± 0.05	0.26 ± 0.06	0.29 ± 0.08	0.29 ± 0.05	0.25 ± 0.02	0.27 ± 0.03	0.25 ± 0.04	0.26 ± 0.05
												

**Note:**

Asterisks indicate sampling months (August, September, October and November).

### Oocyte size, gonad maturity stage and gonadosomatic index

**Figure 2:**
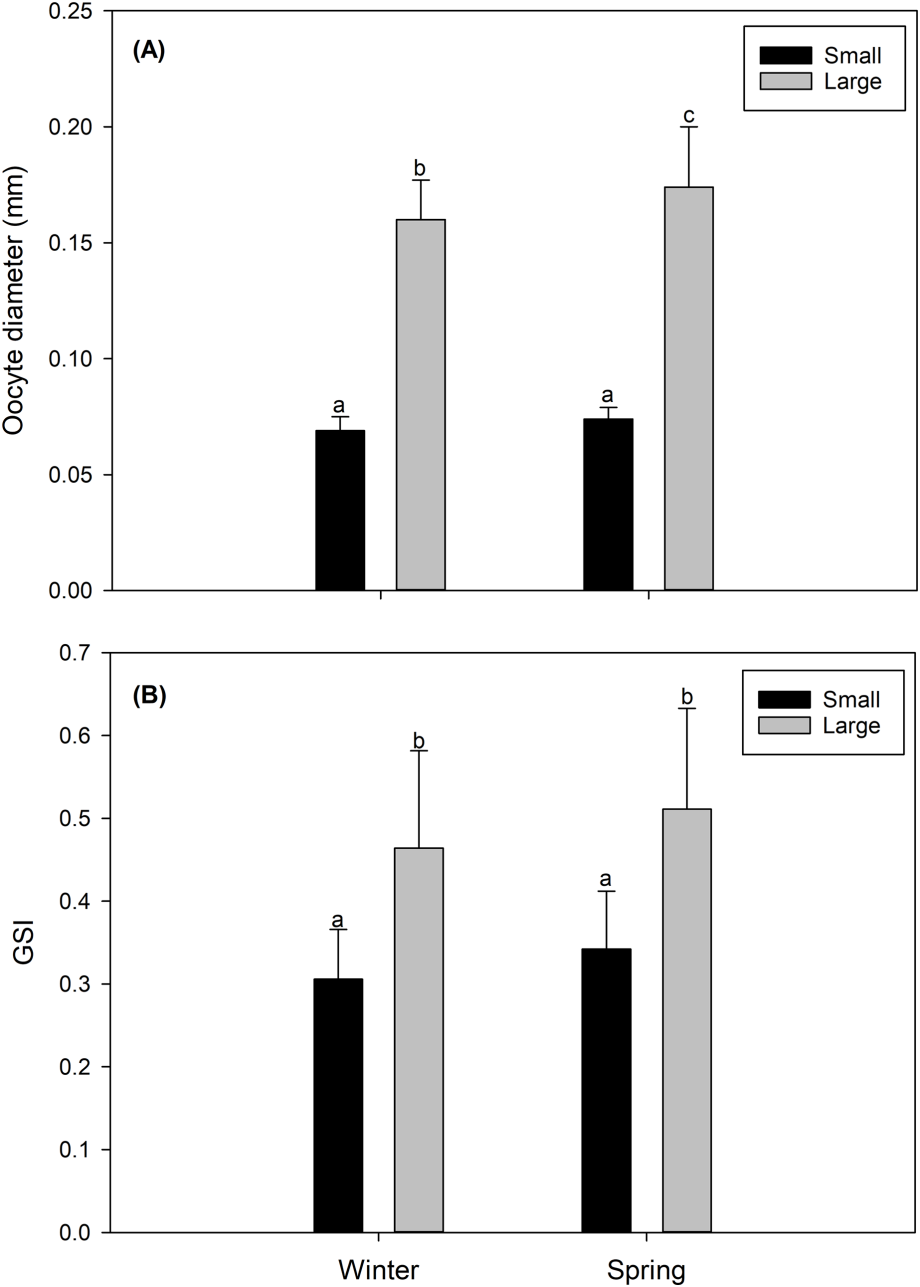
Seasonal variation (winter *vs* spring) (A) in the diameter oocyte (mm) and (B) in the gonadosomatic index (GSI) of the female swordfish of two body size ranges and/or distinct degrees of sexual maturity (small/virginal *vs* large/maturing females). Each bar indicates the average value with its respective standard deviation. For each season and size range (*N* = 11), total *N* = 44. Different letters indicate significant differences (*P* < 0.05).

In both study seasons (winter *vs* spring), the size of the oocyte, and consequently the gonad maturity state and the GSI were more advanced and/or higher in LM females than in SV females. Particularly, LM females, predominantly in their gonads, presented oocytes considered as “maturing” (Data S1) with a size of 0.164 ± 0.013 mm in the winter, and 0.171 ± 0.026 mm in the spring (Fig. 2A). Comparatively, gonads of SV females contained mainly “inactive” oocytes (Data S1), with a size of 0.069 ± 0.006 mm in the winter, and 0.074 ± 0.004 mm in the spring (Fig. 2A). Consistently, the GSI presented significantly higher values in LM females (winter: 0.491 ± 0.037; spring: 0.531 ± 0.066) than in SV females (winter: 0.306 ± 0.06; spring: 0.343 ± 0.055) (Fig. 2B).

### Integrated biochemical condition of the gonad

#### Lipids content

**Figure 3:**
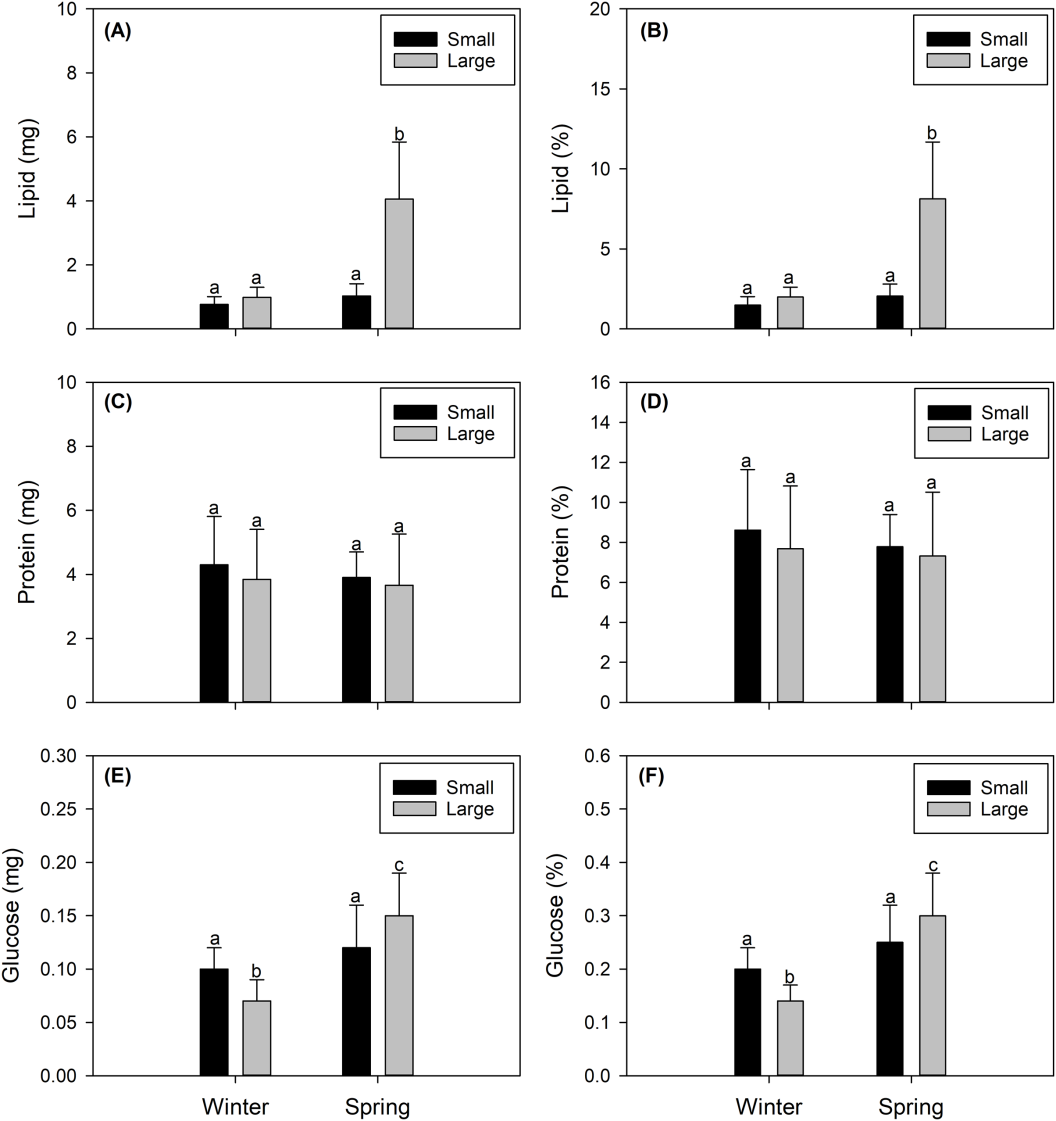
Seasonal variation in the integrated biochemical condition of the gonads of female swordfish. Seasonal variation (winter *vs* spring) in lipid content (A), percentage of lipids (B), protein content (C), percentage of proteins (D), glucose content (E), and percentage of glucose (F) of female swordfish (*Xiphias gladius*) of two body size ranges and/or distinct degrees of sexual maturity (small and/or virginal *vs* large and/or maturing females). Values (A), (C), and (E) are expressed in mg; and values (B), (D), and (F) are expressed in percentage (%) of dry weight. Each bar indicates the average value with its respective standard deviation. For each season and size range (*N* = 11), total *N* = 44. Different letters indicate significant differences (*P* < 0.05).

The total lipid contents found in female *X. gladius* gonads were consistently different when comparing seasons (winter *vs* spring) (ANOVA, F_1,43_ = 53.54; *P* < 0.001) and sizes (ANOVA, F_1,43_ = 51.16; *P* < 0.001) (Table 2). In the spring, the gonads of LM females had higher lipid contents (4.06 ± 1.77 mg) than all other analyzed females. The gonads of SV winter females showed the lowest values (0.773 ± 0.244 mg). While the gonads of the other two treatments showed similar values (*i.e*., SV spring females: 1.027 ± 0.38 mg; LM winter females: 0.994 ± 0.313 mg (Fig. 3A)). Similar tendencies were observed in lipid percentages, with significant differences found between seasons (ANOVA, F_1,43_ = 54.17; *P* < 0.001) and sizes (ANOVA, F_1,66_ = 51.83; *P* < 0.001) (Table 2). LM females analyzed in the spring showed higher lipid percentages (8.127 ± 3.559%) than all other evaluated females. The gonads of SV winter females showed the lowest values (1.491 ± 0.53%). While the gonads of the other two treatments showed similar values (*i.e*., SV spring females: 2.055 ± 0.76%; LM winter females: 1.99 ± 0.627% (Fig. 3B, Data S1).

**Table 2:** Statistical table of the integrated biochemical condition of female swordfish. Results of the two-way ANOVA assessing differences in the contents of lipids (in mg and %), proteins (in mg and %), glucose (in mg and %) and energy (in J mg^−1^) in the gonads of female *Xiphias gladius*, considering the sampled season (“Season”) and female size ranges (“Size”) as fixed factors.

Biochemical parameters	Factor	df	MS	F	*P*
Oocyte diameter (mm)	Season	1	0.0004	1.963	0.169
	Size	1	0.101	452.2	<0.001*
	Season × size	1	0.00002	0.091	0.765
	Total	43	0.003		
GSI	Season	1	0.019	1.953	0.17
	Size	1	0.295	30.08	<0.001*
	Season × size	1	0.0003	0.03	0.864
	Total	43	0.017		
Lipids (mg)	Season	1	1.385	53.54	<0.001*
	Size	1	1.323	51.16	<0.001*
	Season × size	1	0.623	24.1	<0.001*
	Total	43	0.102		
Lipids (%)	Season	1	1.467	54.17	<0.001*
	Size	1	1.403	51.83	<0.001*
	Season × size	1	0.57	21.05	<0.001*
	Total	43	0.105		
Protein (mg)	Season	1	0.937	0.472	0.496
	Size	1	1.335	0.672	0.417
	Season × size	1	0.143	0.072	0.79
	Total	43	1.903		
Protein (%)	Season	1	3.748	0.472	0.496
	Size	1	5.338	0.672	0.417
	Season × size	1	0.571	0.072	0.79
	Total	43	327.2	7.61	
Glucose (mg)	Season	1	0.028	28.7	<0.001*
	Size	1	0.0000007	0.0007	0.979
	Season × size	1	0.008	8.523	<0.01*
	Total	43	0.002		
Glucose (%)	Season	1	0.114	28.7	<0.001*
	Size	1	0.000003	0.0007	0.979
	Season × size	1	0.034	8.523	<0.01*
	Total	43	0.007		
Energy (J mg^−1^)	Season	1	0.216	12.68	<0.001*
	Size	1	0.152	8.922	<0.01*
	Season × size	1	0.191	11.22	<0.01*
	Total	43	0.029		

**Note:**

GSI, Gonadosomatic index. Significant differences (*P* < 0.05) are highlighted with asterisk.

#### Protein content

**Figure 4:**
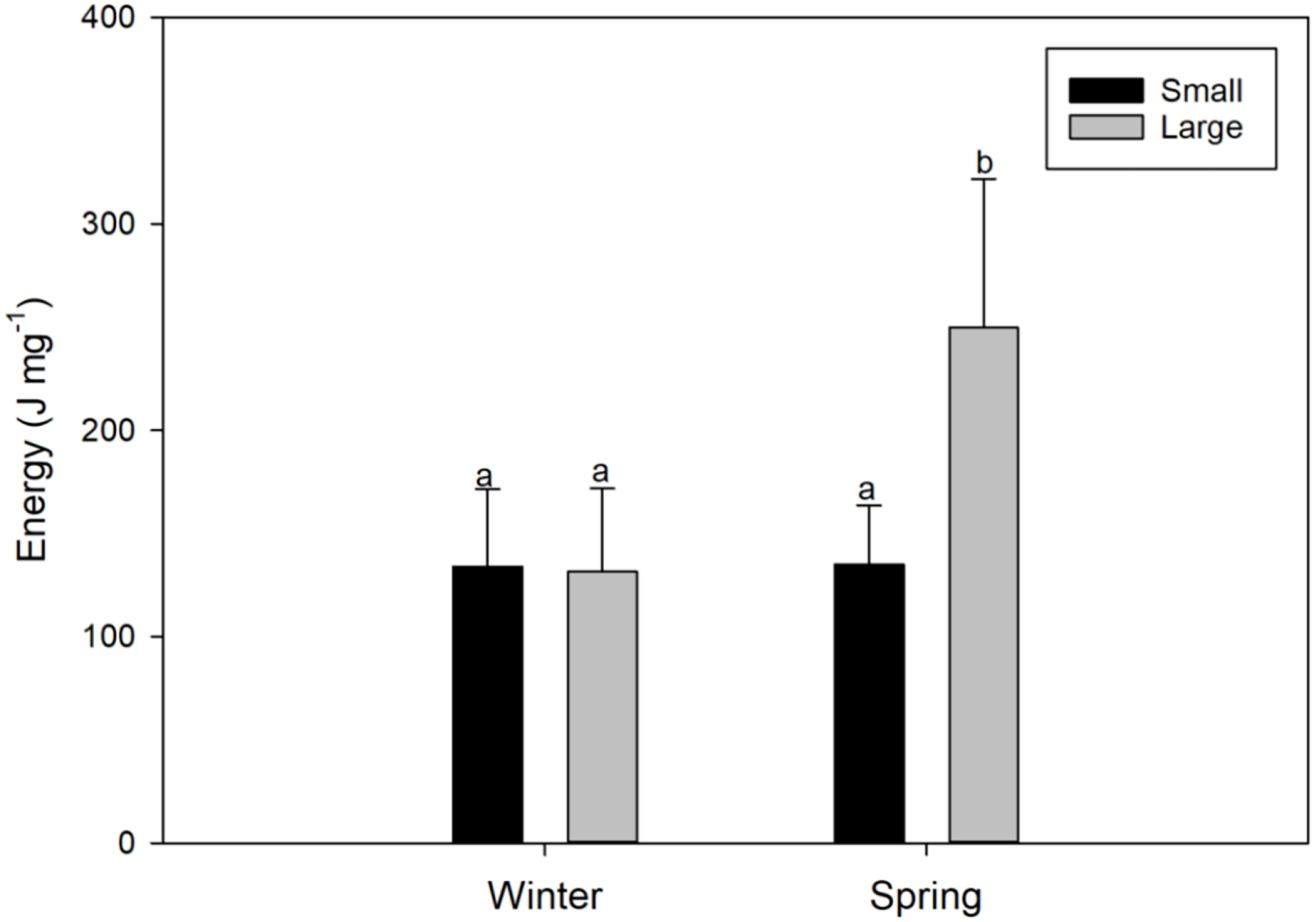
Seasonal variations (winter *vs* spring) in the energy content of female swordfish (*Xiphias gladius*) of two body size ranges and/or distinct degrees of sexual maturity (small and/or virginal *vs* large and/or maturing females). Each bar indicates the average value with its respective standard deviation. For each season and size range (*N* = 11), total *N* = 44. Different letters indicate significant differences (*P* < 0.05).

Protein content showed no significant differences between seasons (ANOVA, F_1,43_ = 0.472; *P* = 0.496) or sizes (ANOVA, F_1,43_ = 0.672; *P* = 0.417) (Table 2). Protein concentrations were around 4 mg for both seasons and size ranges (see Fig. 4C). Similarly, protein percentages showed no significant differences when comparing seasons (ANOVA, F_1,43_ = 0.472; *P* = 0.496) or sizes (ANOVA, F_1,43_ = 0.672; *P* = 0.417), reaching values around 9% (Fig. 3D, Data S1).

#### Glucose content

The glucose content showed a similar trend, varying only slightly with significant differences between seasons (ANOVA, F_1,43_ = 28.7; *P* < 0.001) but no significant differences between sizes (ANOVA, F_1,43_ = 0.0001; *P* < 0.979) (Table 2), reaching from 0.07 to 0.15 mg (Fig. 3E). Glucose percentages were also similar with differences between seasons (ANOVA, F_1,43_ = 28.7; *P* < 0.001) but no differences between sizes (ANOVA, F_1,43_ = 0.0001; *P* = 0.979) (Table 2), varying from 0.14 to 0.3% among the different females (Fig. 3F, Data S1).

#### Energy content

The energy content found in the gonads of female *X. gladius* was significantly different for the two seasons (ANOVA, F_1, 43_ = 12.68; *P* < 0.001) but no for the two sizes range (ANOVA, F_1,43_ = 8.92; *P* = 0.005) (Table 2). LM females analyzed in the spring had significantly higher amounts of energy (249.8 ± 92.29 J mg^−1^) than all of the other analyzed females (*i.e*., LM winter females: 131.3 ± 40.51 J mg^−1^; and SV females: 134 ± 37.31 J mg^−1^ in winter and 134.8 ± 28.85 J mg^−1^, in spring; Fig. 4, Data S1).

### Fatty acid profiles

**Figure 5:**
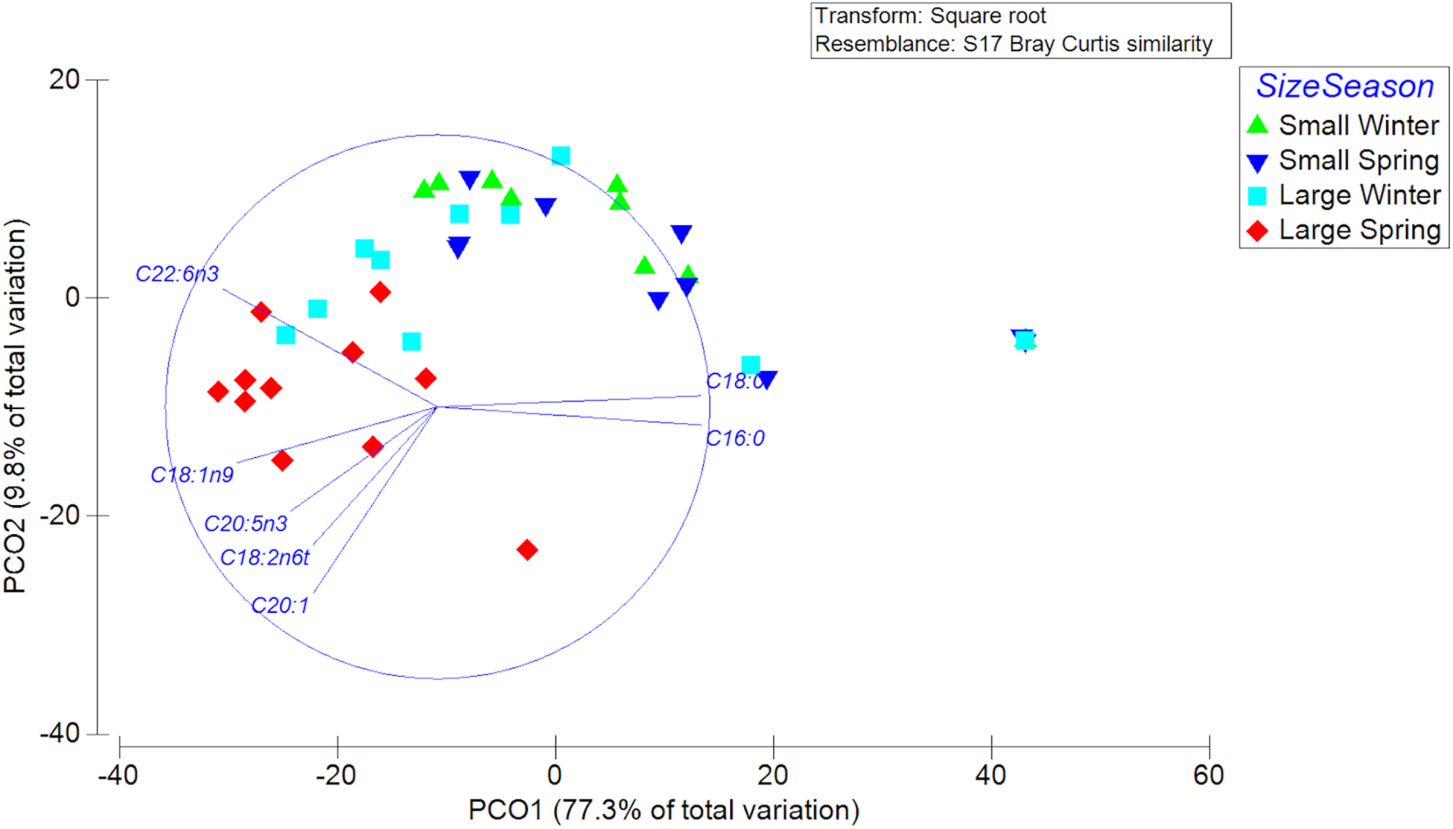
Principle Coordinate Analysis of the fatty acid profile of gonad of female swordfish. The figure shows two different seasons (winter *vs* spring) and two body size ranges and/or distinct degrees of sexual maturity (small and/or virginal *vs* large and/or maturing females), total *N* = 44.

A total of 23 different fatty acids were found in the gonads of female swordfish. The greater diversity of FAs was found in LM female of spring, while the lowest diversity of fatty acids was found in SV female of winter (Table 3; Data S2). The FAs that were detected to have greater abundances (*i.e*., palmitic (C16:0), oleic (C18:1*n*-9), and DHA (C22:6*n*-3)) in the gonads of female *X. gladius* showed significant differences when comparing individuals from both seasons and sizes (Table 3, Data S2). Consistently, the FA profiles varied significantly between seasons (PERMANOVA, pseudo-F: 4.03; *P* < 0.05) and size ranges (PERMANOVA, pseudo-F: 11.278; *P* < 0.01). A SIMPER analysis showed a high similarity between seasons, mainly explained by the high contribution of palmitic (C16:0), stearic (C18:0), oleic (C18:1*n*-9), and docosahexaenoic acid (C22:6*n*-3) (Table 4). For the size factor, the SIMPER shows a high similarity, explained by the high contribution of palmitic (C16:0), stearic (C18:0), oleic (C18:1*n*-9), and docosahexaenoic acid (C22:6*n*-3) (Table 4). In the PCoA, the first axis explained most of the variability between females (77.3%) and the second axis only a minor fraction (9.8%) (Fig. 5).

**Table 3:** Fatty acid of the gonads of female swordfish. Fatty acid profiles (expressed in mg g^−1^ DW and percentage) of the gonads of female *Xiphias gladius* from different seasons (winter *vs* spring) and different size ranges and/or distinct degrees of sexual maturity (small and/or virginal *vs* large and/or maturing).

	Winter		Spring	
Fatty acid	Small	Large	Small	Large
C14:0	n.d.	0.11 ± 0.02^a^ (2.36)	n.d.	0.63 ± 0.61^a^ (1.72)
C15:0	n.d.	n.d.	n.d.	0.21 ± 0.06 (0.57)
C16:0	0.52 ± 0.33^a^ (20.7)	0.76 ± 0.53^a^ (15.8)	0.74 ± 0.51^a^ (23.36)	5.52 ± 3.72^b^ (15.06)
C17:0	0.15 ± 0.04^a^ (5.82)	0.12* (2.51)	0.12 ± 0.03^a^ (3.8)	0.31 ± 0.17^b^ (0.84)
C18:0	0.31 ± 0.16^a^ (12.49)	0.39 ± 0.23^a^ (8.18)	0.49 ± 0.29^a^ (15.51)	2.64 ± 2.07^b^ (7.22)
C21:0	n.d.	n.d.	n.d.	0.18* (0.5)
C23:0	n.d.	n.d.	n.d.	0.88 ± 0.52 (2.4)
C24:0	n.d.	n.d.	n.d.	0.2 ± 0.08 (0.55)
∑ SFA	0.98 ± 0.27^a^ (39.01)	1.38 ± 0.44^b^ (28.86)	1.35 ± 0.43^b^ (42.67)	10.57 ± 2.72^c^ (28.87)
C14:1	n.d.	n.d.	0.14 ± 0.03^a^ (4.42)	0.07 ± 0.01^b^ (0.2)
C16:1	0.14 ± 0.06^a^ (5.67)	0.2 ± 0.06^a^ (4.08)	0.15 ± 0.04^a^ (4.79)	0.96 ± 0.71^b^ (2.61)
C17:1	n.d.	n.d.	n.d.	0.27 ± 0.13 (0.74)
C18:1*n*9	0.39 ± 0.3^a^ (15.87)	1.08 ± 0.69^b^ (22.52)	0.74 ± 0.67^ab^ (23.34)	9.85 ± 8.07^c^ (26.88)
C20:1	n.d.	0.24 ± 0.11^a^ (5.05)	n.d.	1.67 ± 1.29^b^ (4.55)
C22:1	n.d.	n.d.	n.d.	0.38 ± 0.13 (1.03)
C24:1	n.d.	n.d.	n.d.	0.24 ± 0.09 (0.67)
∑ MUFA	0.53 ± 0.27^a^ (21.54)	1.52 ± 0.62^b^ (31.65)	1.03 ± 0.55^ab^ (32.55)	13.44 ± 5.44^c^ (36.69)
C18:2*n*6t	n.d.	n.d.	n.d.	0.22 ± 0.2 (0.61)
C18:2*n*6c	n.d.	n.d.	n.d.	0.19 ± 0.04 (0.51)
C18:3*n*6	n.d.	n.d.	n.d.	0.28 ± 0.17 (0.76)
C20:2*n*6	n.d.	n.d.	n.d.	0.08* (0.23)
∑ PUFA *n*6	n.d.	n.d.	n.d.	0.77 ± 0.16 (2.11)
C18:3*n*3	n.d.	n.d.	n.d.	0.2 ± 0.03 (0.56)
C20:3*n*3	0.36 ± 0.21^a^ (14.59)	0.16 ± 0.02^b^ (3.31)	0.22 ± 0.03^c^ (6.85)	0.9 ± 0.65^d^ (2.45)
C20:5*n*3	n.d.	0.26 ± 0.04^a^ (5.43)	n.d.	1.42 ± 1.32^b^ (3.87)
C22:6*n*3	0.62 ± 0.53^a^ (24.85)	1.47 ± 0.72^b^ (3.75)	0.57 ± 0.43^a^ (17.92)	9.53 ± 9.01^c^ (25.46)
∑ PUFA *n*3	0.98 ± 0.46^a^ (39.44)	1.89 ± 0.83^b^ (39.49)	0.79 ± 0.38^a^ (24.77)	12.05 ± 6.38^c^ (32.33)
∑ PUFA	0.98 ± 0.46^a^ (39.44)	1.89 ± 0.83^b^ (39.49)	0.79 ± 0.38^a^ (24.77)	12.82 ± 5.67^c^ (34.44)
Total FA	2.49 ± 0.33^a^	4.79 ± 0.62^b^	3.17 ± 0.46^c^	36.83 ± 4.65^d^

**Note:**

SFA, Saturated fatty acid; MUFA, Monounsaturated fatty acid; PUFA, Polyunsaturated fatty acid (*n*-3 and *n*-6). Total SFA: sum of C8:0, C12:0, C13:0, C14:0, C15:0, C16:0, C17:0, C18:0, C20:0, C21:0, C22:0 and C24:0; Total MUFA: sum of C14:1*n*-5, C16:1*n*-7, C18:1*n*-9, C20:1*n*-9, C22:1*n*-9 and C24:1*n*-9; Total PUFA n-6: sum of C18:2*n*-6c, C18:3*n*-6, C20:2*n*-6, C20:3*n*-6; Total PUFA *n*-3: sum of C18:3*n*-3, C22:6*n*-3, C20:3*n*-3, C20:5*n*-3; Total PUFA: sum of PUFA *n*-3 and *n*-6; Total FA: sum of Total SFA, Total MUFA and Total PUFA. n.d., no detected. An asterisk indicates that the standard deviation could not be obtained. Different superscript letters indicate significant differences. The percentage is indicated in parentheses, and the mean values ± SD are provided.

**Table 4:** Similarity percentage analysis (SIMPER) of the female swordfish. The table shows the contribution of each fatty acid (FA) identified among females of different size ranges and/or distinct degrees of sexual maturity (small and/or virginal *vs* large and/or maturing females) collected during two different seasons (winter *vs* spring).

Group	Average similarity	FA	Av. abund.	Av. sim.	Sim/SD	Contrib. %	Cum. %
Small	72.43	Palmitic	5.98	27.23	3.58	37.59	37.59
		Stearic	4.84	21.28	3.32	30.21	67.8
		Oleic	3.58	11.37	1.14	15.69	83.49
		DHA	3.3	9.21	0.98	12.72	96.21
Large	73.39	Palmitic	4.79	17.8	2.76	24.26	24.46
		Oleic	4.72	15.15	2.05	20.64	44.9
		Stearic	3.48	12.5	2.19	17.03	61.93
		DHA	4.27	11.58	1.27	15.79	77.72
Winter	69.98	Palmitic	5.66	25	3.08	35.72	35.72
		Stearic	4.44	18.99	2.56	27.13	62.86
		Oleic	3.56	11.09	1.14	15.85	78.7
		DHA	3.82	10.63	0.99	15.19	93.89
Spring	75.84	Palmitic	5.11	20.03	2.47	26.41	26.41
		Oleic	4.74	15.42	2.04	20.34	46.75
		Stearic	3.88	15.39	2	20.3	67.05
		DHA	3.75	10.17	1.33	13.41	80.45

**Note:**

DHA, Docosahexaenoic acid; AV. abund., average abundance; Av. Sim., average similarity; Sim/SD, standard deviation of similarity; Contrib. %, contribution; Cum. %, cumulative contribution.

## Discussion

The present study revealed variations in the biochemical condition of gonads of the swordfish *Xiphias gladius* from the SEPO. These variations may be related to reproductive energetic processes, habitat adaptations, and seasonal changes in key environmental parameters, such as temperature and food availability (Espinoza & Bertrand, 2014; Guzmán-Rivas et al., 2021; Hernández-Santoro, Landaeta & Castillo Pizarro, 2019; Koenigstein et al., 2016; Parada et al., 2013). They are also pivotal to explain population dynamics and stability, especially those regarding reproduction, recruitment, and offspring survival (Jakobsen et al., 2016). These processes greatly affect adult populations that are exploited by commercial fisheries in the SEPO (Barría et al., 2020).

In large marine fishes, females tend to be long-lived individuals (Heppell et al., 2005). In this context, after several reproductive events, the older and larger females tend to produce larger offspring than younger females and/or those females that are in their first reproductive period (Cole, 2010; Evans & Claiborne, 2005; Jakobsen et al., 2016). Among the main findings of our study, large and/or mature female swordfish had a greater amount of lipids in their gonads than small and/or virginal females. In this case, we reveal that large female swordfish present higher GSI and allocate most of their energetic reserves to the gonads to subsequently generate larger oocytes. While the small females probably invest their energy budget into a substantial part for growth and another minor for reproduction, this characterized with low GSI values and small oocytes, as shown in the present study. Future studies are necessary to evaluate the energetic balance of female fish and develop a bioenergetic model considering all female sizes (*i.e*., small, large and very large). Such information could aid substantially to understanding how the size of females affects the quality and condition of their offspring.

Regarding the biochemical composition of the gonads of female swordfish in our study, the protein and glucose contents did not differ between seasons or size ranges. Interestingly, when comparing these biochemical constituents with previous studies carried out in the muscle of adult swordfish individuals (Lazo-Andrade et al., 2021a), they presented seasonal variations. Probably, these opposite trends and differences in the content of these biomolecules between these tissues and/or organs are related according to their functionality and degree of use in these tissues (*i.e*., muscle, gonad) for fundamental physiological processes (*e.g*., growth and reproduction) (Eliassen & Vahl, 1982; Evans & Claiborne, 2005; Hochachka & Somero, 2002). The slight fluctuation in the protein content per gonad between seasons may be explained by the requirement to maintain protein concentrations to form structural components during growth (Cole, 2010; Hochachka & Mommsen, 1995). Similar results have been previously described in cold-temperate marine fishes (Hochachka & Mommsen, 1995). Furthermore, the glucose contents of gonads remained relatively constant for the two evaluated seasons and size ranges. This most likely occurs because glucose is the primary source of energy (immediate fuel) compared to proteins (structural component) and lipids (energy reservoir) (Hochachka & Mommsen, 1995). Therefore, glucose may be allocated to the gonads in relatively conservative amounts because it can be easily replenished.

In contrast, lipid contents and energy of female swordfish greatly varied when comparing the two studied seasons (winter *vs* spring) and two body sizes ranges and/or distinct degrees of sexual maturity (small and/or virginal *vs* large and/or maturing). These variations may be related to the metabolism of vitellogenin, an important sex hormone for fish reproduction, as well as for the survival of offspring before their first exogenous feeding (Cole, 2010). Similar seasonal patterns of lipid and vitellogenin contents have been described in mollusks and highly migratory fishes (Arts, Brett & Kainz, 2009; Cole, 2010). The sequence and rates of use of these biochemical components (proteins, glucose, and lipids) is reflected in a consistent manner in variations in the energy content, mainly explained herein by the contribution of lipids among large females evaluated in the spring.

The FA profiles showed that DHA is present in greater proportion among large females that were evaluated in the winter. This essential FA must be assimilated by the female through food and then transferred from the gonads into the oocytes (Arts, Brett & Kainz, 2009; Sargent, 1995; Steinberg, 2018). Recent studies on fish reproduction and development have shown that DHA is pivotal for neuronal development and the formation of nervous tissue during embryogenesis (Arts, Brett & Kainz, 2009; Hou & Fuiman, 2020; Steinberg, 2018). It has been demonstrated that female swordfish obtain large amounts of DHA from planktonic food in the summer and then transfer it to their offspring, which consume DHA within, approximately, the first 7 days after hatching (Abascal et al., 2009; Cerna, 2009). Palmitic acid (C16:0), stearic acid (C18:0), and oleic acid (C18:1*n*9) were common among large females assessed in the spring. These FAs can maximize fecundity under optimal environmental conditions (*i.e*., temperature and food availability). These FAs are of utmost importance as a source of metabolic energy during the growth and formation of eggs. Smida, Marzouk & Cafsi (2009) analyzed the profiles of FAs in the gonadal tissue of swordfish from the Mediterranean Sea and found about 5% of FAs in the gonad relative to the sample’s wet weight. Smida, Marzouk & Cafsi (2009) also found that palmitic acid (C16: 0) (17.08–26.9%) was the most abundant saturated fatty acid in the gonad. In turn, oleic acid (C18:1*n*-9) was found to be the most abundant monounsaturated FA (*i.e*., 13.3–35.5%) and DHA (C22:6*n*-3) the most abundant (*i.e*., 14.5–28.8%) polyunsaturated FA. All of these FA values are similar to those found in our study.

Our results regarding the FA profiles of female gonads of *X. gladius* confirm the role that swordfish play as a top predator in the marine food web of the SEPO. For example, the large proportion of saturated FAs (*e.g*., palmitic (C16: 0) and stearic (C18: 0)) FAs could be linked principally to the consumption of pelagic prey (*e.g*., horse mackerel and sawfish), while the presence of polyunsaturated FAs (*e.g*., oleic (C18: 1*n*-9) and docosahexaenoic (C22: 6*n*-3)) FAs could be related to the consumption of planktivorous fishes (*e.g*., sardines), as well as demersal species (*e.g*., squids, hake, squat lobsters, and myctophids). A similar feeding and trophodynamic pattern, analyzed using FAs profile as trophic markers (for concept see: Dalsgaard et al. (2003), Pethybridge et al. (2018)), has been recorded in top, migratory predator species (*e.g*., swordfish, marlin, and tuna) that inhabit coastal and oceanic temperate areas (Pethybridge et al., 2010; Pethybridge et al., 2015; Smida, Marzouk & Cafsi, 2009). Consistently, a similar trend of higher amounts of FAs in spring, characterized by a greater diversity of PUFAs (present study done in gonads), was also reported in swordfish muscle by Lazo-Andrade et al. (2021a).

The swordfish is a highly migratory species with a wide global distribution (Mejuto & García-Cortés, 2014; Nakamura, 1985; Stoehr et al., 2018). Hence, this species shows a great variety of prey, from small coastal pelagic fishes to large oceanic animals, such as squids (Abid et al., 2018; Trujillo-Olvera et al., 2018; Zambrano-Zambrano et al., 2019). Therefore, its biochemical composition, energetic content, and FA profiles can vary, not only temporarily, according to seasonal changes, but also spatially with latitude, among populations, and/or geographic regions (Meyer et al., 2019). Therefore, future studies should consider how the bioenergetic condition of this species varies within its wide ecological and latitudinal distribution.

In our findings, the criteria indicative of the sexual maturity of female swordfish (body size and size at first sexual maturity) were found to be key parameters that can modulate the size of the oocytes and the gonadosomatic index (GSI). In particular, small female virginal swordfish presented mostly inactive oocytes and low GSI values in their gonads, while active oocytes and high GSI values predominated in large maturing females. A similar trend in the degree of sexual maturity as a function of body size has also been recorded in individuals of the same species from other geographical regions (DeMartini, Uchiyama & Williams, 2000; Mejuto & García-Cortés, 2014; Poisson & Fauvel, 2009), and also for other species of fish considered highly migratory (tunas, marlins, common dolphinfish: Chang et al. (2018), Shimose et al. (2016)).

In turn, the quality of gonads may change, not only in relation to the degree of maturity of the individuals, but also throughout the year due to variations in the environmental conditions experienced by females in the habitat (Howells et al., 2017). Thus, a cascade effect could occur, affecting the condition and quality of the oocytes, which then affect the entire life cycle of the species, including the survival and development of both juveniles and adult individuals (Faleiro & Narciso, 2010). Therefore, ample information regarding changes in the quality of the gonads in relation to female sizes and seasons is key to establish effective management of this fishery resource, and could help in decision-making processes regarding the protection of female breeding swordfish and their potential offspring (Bucholtz et al., 2013; Faleiro & Narciso, 2010).

Regulations among highly migratory fishery resources are inherently complicated because the international cooperation of Southeastern Pacific countries is required, together with national regulations (Barría et al., 2020). In this context, the use of FAs has been steadily increasing as a conventional tool for researchers focused on trophic interactions, food webs, and biomarker of migratory species in the SEPO (Lazo-Andrade et al., 2021a; Parrish et al., 2015; Wang et al., 2019). Biological and ecological data has also been shown to be a reliable tool capable of thoroughly analyzing the trophic ecology of oceanic predators (Meyer et al., 2019; Pethybridge et al., 2010; Young et al., 2015). In addition, FAs can be used as indicators of the offspring condition and that of the subsequent recruits to populations (Hasek & Felder, 2005). FAs can also be used as potential indicators of the individual nutritional and energetic condition throughout the year. In this context, in our study and as reported in teleost fish (Tocher, 2003), long-chain polyunsaturated essential fatty acids (EPA: C20:5*n*3; DHA: C22:6*n*3) and some of their precursors (Monroig et al., 2022) abundantly recorded in the gonads of large female swordfish during spring (present study), can be used as an appropriate biological indicator to verify gonadal maturation, as well as an indicator of metabolism and/or nutrient requirements during the breeding season (Steinberg, 2022). Considering a holistic perspective, we herein showed that seasonal variations in the biochemical condition and body size are pivotal parameters to develop models of energetic budgets and predict responses of highly migratory species to environmental differences. The information provided by our study could be used to preserve commercial fishing stocks and integrative conservation management plans that promote the sustainable exploitation of resources.

Finally, because *X. gladius* presents several reproductive events throughout its long-life, with a late maturation (at 4 years of age) and a high observed longevity (16 years), future studies should consider not only comparisons between virginal *vs* maturing females (like the present study), but also specimens of long-lived females (called “mega-spawners”, (Sierra Castillo & Fujiwara, 2021)) to elucidate whether the quality of the gonad of long-lived females can contribute to the production of active and/or viable oocytes. In addition, because other organs (*i.e*., the liver) play a fundamental role in reproduction (*i.e*., in the storage and subsequent transfer of energy to the gonad for oocyte formation), at the individual level, it would be interesting to identify how the dynamics of the energy reserves in different organs (liver, gonad, muscle) and at different stages of sexual maturity and/or age of female swordfish during its life-cycle (virginal, mature spawners, mega-spawners) affect fishing management and exploitation in the SEPO.

## Conclusion

The lipid contents and overall energy content was highest in large females evaluated in the spring. The protein and glucose contents were similar in the two assessed female sizes and seasons. Fatty acid profiles varied between the two female sizes and seasons examined. The FAs C16:0, C18:0, C18:1*n*9, and C22:6*n*3 were the main contributors to the variability between winter and spring. These results may be related to reproductive energetic processes, adaptations to habitat, and also to the variability and seasonality of key environmental factors that occur in temperate latitudes of the Southeastern Pacific Ocean. These variations are fundamental to explain population and process dynamics (*e.g*., reproduction, recruitment and offspring survival), and are therefore pivotal for stock evaluation and for generating integrative fishery management models.

## Supplemental Information

10.7717/peerj.15524/supp-1 Supplemental Information 1 Morphometric and biochemical parameters of female swordfish. Catch data (ship, year of capture, sample code and morphometric parameters) and biochemical data (protein, glucose, lipids and energy) of female swordfish.

10.7717/peerj.15524/supp-2 Supplemental Information 2 Fatty acids profile of female swordfish. The fatty acids profile of gonads of female swordfish in two different seasons (winter and spring) with two different size (small and large).

10.7717/peerj.15524/supp-3 Supplemental Information 3 Oocytes of the swordfish. The oocytes of (A) small female swordfish and (B) large female swordfish.

## Additional Information and Declarations

### Data Availability

The following information was supplied regarding data availability: The raw measurements are available in the Supplemental Files.
