# Supplementary figures and images for: Temporal and inter-individual changes in the integrated biochemical condition of the gonads of female swordfish (Xiphias gladius) from the Southeastern Pacific Ocean
Source: PeerJ. 2023 Jun 6;11:e15524. doi: 10.7717/peerj.15524 (PMC10252824; doi:10.7717/peerj.15524)

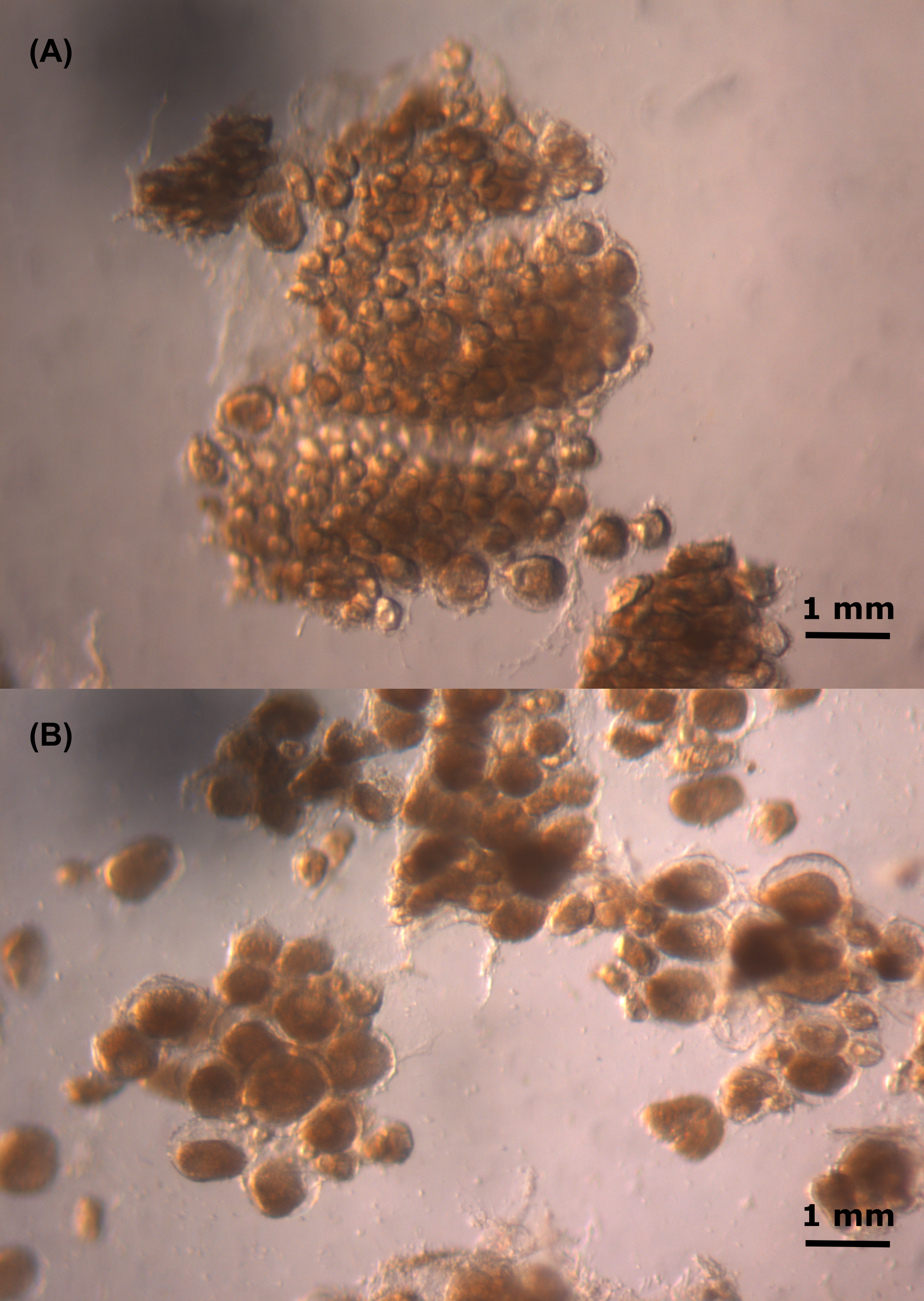

Supplement: Supplemental Information 3 — The oocytes of (A) small female swordfish and (B) large female swordfish. [file peerj-11-15524-s003.png]
